# Genome Sequences of the Lignin-Degrading Pseudomonas sp. Strain YS-1p and Rhizobium sp. Strain YS-1r Isolated from Decaying Wood

**DOI:** 10.1128/genomeA.00019-15

**Published:** 2015-03-05

**Authors:** Madhu Prabhakaran, Matthew B. Couger, Colin A. Jackson, Tyler Weirick, Babu Z. Fathepure

**Affiliations:** a Department of Microbiology and Molecular Genetics, Oklahoma State University, Stillwater, Oklahoma, USA; b Department of Biochemistry and Molecular Biology, Oklahoma State University, Stillwater, Oklahoma, USA

## Abstract

Pseudomonas sp. strain YS-1p and Rhizobium sp. strain YS-1r were isolated from a lignin-degrading enrichment culture. The isolates degraded lignin-derived monomers, dimers, alkali lignin, and, to a smaller extent (3% to 5%), lignin in switch grass and alfalfa. Genome analysis revealed the presence of a variety of lignin-degrading genes.

## GENOME ANNOUNCEMENT

Lignin is the second most abundant terrestrial polymer after cellulose and is found in most terrestrial plants in significant amounts ranging from 15% to 40% of dry weight (1). Lignin cross-links with hemicellulose and cellulose and resists microbial attack forming a major roadblock for effective saccharification of plant biomass (2, 3). To date, studies have focused primarily on fungal ability to breakdown lignin as they produce high levels of efficient enzymes that degrade lignin (4–6). However, in recent years, emphasis has shifted toward understanding the bacterial role in lignin degradation because bacteria are widespread and can offer novel catalytic pathways for the development of efficient pretreatment technologies (7–11). The primary goals of this study were to isolate and characterize bacteria that degrade lignin as the sole source of carbon. We have isolated a Pseudomonas sp. strain YS-1p and Rhizobium sp. strain YS-1r that degrade lignin and lignin-like compounds as the sole sources of carbon from an enrichment developed from a decaying wood from a thermal pond at Yellowstone National Park, WY. To obtain insights into the lignin-degradation capacity of strains, YS-1p and YS-1r, draft genomes were analyzed.

Genomes of the strain YS-1p and strain YS-1r were sequenced using the Illumina miSeq platform using 250×2 paired-end reads. Sequence libraries were created using the standard Illumina Tru-seq library creation kit with an average insert size of 550 bp. Quality filtered sequence data were subsampled to ~150× coverage and assembled with the short read de Brujin graph (12) assembly program Velvet (13) using a k-mer value of 55. This assembly produced 103 contigs with a contig *N*_50_ of 199 kb, and total 6,380,264 bp for strain YS-1p and similarly the assembly produced 84 contigs with a contig *N*_50_ of 186 kb, and a total 6,385,152 bp for strain YS-1r. Genes were identified using the Prodigal algorithm (14). From the resulting assemblies, 5,796 gene models were produced for strain YS-1p and 5,709 for YS-1r. All proteins sequences were functionally annotated using a combination of NCBI Blast (15) and HMMER 3.0 (16) against the PFAM 27.0 database (17).

Genome analysis revealed that both organisms contain genes that code for enzymes needed for the degradation lignin and lignin-derived aromatic compounds including laccase, Dyp-peroxidase, beta-etherase, vanillate *O*-demethylase, feruloyl esterase, carboxyl esterase, cytochrome P450, and chloroperoxidase. Also, genes for aromatic ring-oxidation and ring-cleavage including phenol 2-monooxygenase, 4-hydroxybenzoate 3-monooxygenase, catechol 2,3-dioxygenase, protocatechuate 3,4-dioxygenase, and gentisate 1,2-dioxygenase were detected. Our laboratory experiments revealed that both isolates can degrade a variety of lignin monomers and dimers as the sole sources of carbon. Also, the organisms utilized alkali lignin (Sigma-Aldrich) and lignin in switch grass and alfalfa as the growth substrates. These observations suggest that bacteria can play important roles in plant biomass conversion technologies.

### Nucleotide sequence accession numbers.

The draft genome sequences of YS-1p and strain YS-1r have been deposited at DDBJ/EMBL/GenBank under the accession numbers JPYP00000000 and JPYQ00000000, respectively. The versions described here are versions JPYP01000000 and JPYQ01000000, respectively.
